# The Role of miRNAs as New Molecular Biomarkers for Dating the Age of Wound Production: A Systematic Review

**DOI:** 10.3389/fmed.2021.803067

**Published:** 2022-01-14

**Authors:** Stefania De Simone, Elena Giacani, Maria Antonella Bosco, Simona Vittorio, Michela Ferrara, Giuseppe Bertozzi, Luigi Cipolloni, Raffaele La Russa

**Affiliations:** ^1^Department of Clinical and Experimental Medicine, Section of Legal Medicine, University of Foggia, Foggia, Italy; ^2^Department of Anatomical, Histological, Forensic and Orthopedic Sciences, Sapienza University of Rome, Rome, Italy

**Keywords:** wound-age estimation, wound-age determination, postmortem miRNA, miRNA, post-mortem investigation

## Abstract

**Background:**

The timing of wounds production is a significant issue in forensic pathology. Although various methods have been evaluated, obtaining an accurate dating of lesions is still a challenge. The pathologist uses many parameters to value wound age, such as histological and immunohistochemical. In recent years, there have been many studies regarding the use of miRNAs in wound-age estimation; indeed, miRNAs have multiple potential uses in forensic pathology.

**Scope:**

This review aims to verify the efficacy and feasibility of miRNAs as a tool for determining the timing of lesions.

**Materials and Methods:**

The authors conducted the systematic review according to the Preferred Reporting Items for Systematic Reviews and Meta-Analysis (PRISMA) guidelines. PubMed was used as a search engine to find articles published between January, 1st 2016 and October, 1st 2021, to evaluate the current state of the art regarding wound-age estimation.

**Results:**

A total of 256 articles were collected; after screening according to PRISMA guidelines, the systematic review included 8 articles. The studies included in this review were all Original articles evaluating the use of biomarkers for wound-age determination.

**Discussion and Conclusion:**

The literature review showed that analysis of miRNA is an innovative field of study with significant potentiality in forensic pathology. There are few studies, and almost all of them are at an early stage. The challenge is to understand how to standardize the samples' selection to obtain reliable experimental data. This observation represents a necessary prerequisite to planning further clinical trials.

## Introduction

The timing of wounds production is a significant issue in forensic pathology. Although various methods have been evaluated, obtaining an accurate dating of lesions is still a challenge (1).

After an injury, the pathologists must consider many vital processes (e.g., hemorrhage, inflammatory cells migration, infiltration, development of granulation tissue) for an accurate wound-age estimation (2).

Morphological and macroscopic analysis was one of the first methods used (3). But it is not of practical use because it cannot be standardized.

Histological evaluation (with Haematoxylin-Eosin staining) can detect the presence of inflammatory cells or substances secreted during the inflammatory process (4). Different types of inflammatory cells reach a peak with exact timing, allowing age estimation of the lesion [neutrophilic granulocytes reach the maximum levels after 24 h (4, 5); macrophages after 2-3 days (4); lymphocytes at least 20 h; fibroblastic cells after 6 days or more (4)].

Immunohistochemical methods can also detect different substances secreted during the inflammatory process (6) [esterase and adenosine triphosphatase increase 1 h after injury; aminopeptidase reaches a peak at about 2 h; acid phosphatase at 4 h; alkaline phosphatase at 8 h (7)] or analyze the expression of Fibronectin, CD62p, and Factor VIII, which are markers involved in coagulation and inflammation processes. These, significantly increase in wounds produced close to death (15–30 min old) (8). Also, the number of matrix metalloproteinase-2 (+) macrophages increases in accordance with wound ages (9).

Histamine is a mast cell degranulation product, used as a marker in forensics (e.g., for asphyxiation) (10). Histamine levels in the skin vary appreciably after wounding; these levels increase significantly between 5 min and 3 h after trauma and decrease until 24 h. The transient infiltration of mast cells occurs in the dermis for 3 h upon vital lesions and is followed by a protracted decrease until 24 h (11, 12).

The real-time polymerase chain reaction (PCR) measures mRNA levels (13). PCR can detect the mRNA levels of inflammatory cytokines and wound-healing factors [e.g., caspase level 3, 8, and 9 expressions in tissues (14); VEGF and TGFb1 mRNA have high grades from the early stages, reaching the peak at day 7 (5)]. As mRNA is not very stable due to the action of ribonucleases (15), it is not very useful for forensic purposes. The stability, moreover, presents significant differences depending on the tissue analyzed: skeletal muscle, heart, and brain are more stable than the pancreas (13).

According to the studies on this topic a single parameter is not sufficient for estimating wound-age; indeed, combining parameters can reduce error.

Therefore, in recent years, many studies have been performed regarding the use of miRNAs in wound-age estimation. Indeed, the researchers found microRNAs (miRNAs) expression at 0, 24, and 48 h after death. Especially high levels of miRNA-205 and miRNA-21 24 h after death were observed (16).

MiRNAs were first described in 1993 by Lee et al. (17), and the term microRNA was born in 2001 (18).

MiRNAs are small non-coding RNA molecules containing about 21–25 nucleotides (19) that interact with the 3′ untranslated region (3′ UTR) of target mRNAs to induce their degradation. They can also interact with the 5′ UTR region, coding sequence, gene promoters and activate translation or regulate transcription (20).

The interaction of miRNAs with their target genes is dynamic. It depends on many factors, such as the subcellular location of miRNAs, and both the abundance of miRNAs and target mRNAs. miRNAs can also be secreted into extracellular fluids and operate as chemical messengers to mediate intercellular communication (20).

About 1-3% of the genome encodes miRNAs, which regulate more than 30% of protein-coding genes (17). Many studies have demonstrated that miRNAs play a significant role in biological processes, including cell proliferation, differentiation, apoptosis, organ development, pathogenesis, metabolic control, and antiviral defense (21).

However, the cellular localization of miRNAs is still widely unappreciated (22).

For this reason, many studies have been carried out in recent years to identify some tissue-specific miRNAs (21, 22). MiRbase, the bioinformatic database of miRNA sequences, reports the 1917 miRNAs discovered and encoded by the human genome [www.mirbase.org].

Microarray or Next Generation Sequencing (NGS) techniques can achieve the analysis of miRNAs. After their identification, the researcher can perform the assay by Real-Time quantitative PCR (qRT-PCR) (23).

Modification in the normal miRNA expression pathway can affect normal cellular physiology and lead to different pathologies.

Chronic lymphocytic leukemia was the first human disease known to be related to miRNA deregulation. Human cancer can be associated with changes in miRNA expression by deregulation of oncogenes and oncosuppressor genes (24–26).

MiRNAs' dysregulation is also involved in other diseases like cardiac hypertrophy and heart failure (27–30), chronic kidney disease (31), obesity (32), and various neurological and neuropsychiatric disorders (33).

The aim of this review is to verify the usefulness of miRNAs as a tool for determining the age of lesions. In our opinion it is necessary to analyze the current state of the art on wound-age estimation before to plan additional experimental studies on these topics.

MiRNAs have multiple potential uses in forensic pathology. In fact, unlike mRNA, they are less sensitive to degradation (due to environmental factors, such as UV light or heat, and the action of cellular ribonucleases) (34) and more stable than mRNA. They can also be extracted together with the DNA profile, representing a double test, useful in case of shortage of biological material (35).

MiRNAs are helpful for the recognition of biological fluids found at a crime scene. Nowadays, only a few miRNAs are recognized as specific for a single biological fluid (especially for blood and seminal fluid, while miRNAs specific for venous and menstrual blood are yet to be determined) (36).

Many authors focalized their research on the study of wound vitality (37). Some authors have focused on differentiating ante- and post-mortem lesions through miRNAs (38), but studies are still few and at an early stage.

Determining the age of production of the wounds is a relevant topic to forensic purposes (39). Over the years, many authors studied how putrefaction affects the concentration of proteins, DNA and RNA (40–43). Maiese et al. (44) analyzed the progress made in recent years, showing that some miRNAs are stable and reasonably reliable as markers. Wang et al. (45) demonstrated that three miRNAs (miR-122, miR-150, miR-195) are stable in the first 24 h of PMI (Post-Mortem Interval), declining after that time. Some studies remarked whether the animal died during the day or at night, considering the modification of other miRNAs (miR-541 and miR-142-p) (46).

MiRNAs have an essential role in moderating cellular adaptations occurring in case of drug abuse and addiction (47). The alteration of regular miRNAs expression occurs even in response to substances' abuse, such as nicotine, cocaine, morphine, and alcohol (48–50).

A further field of application for miRNAs is as anti-doping marker (51, 52).

An innovative field of application for miRNAs is the estimation of wound-age, as miRNAs are variously involved in wound healing physiology and physiopathology from injury to re-epithelialization (53, 54).

Aunin et al. (55) investigated the expression of miR-200 in skin wounds of subjects of different ages, marking its role in wound repair. The study identified miR-200c as a critical determinant that inhibits cell migration during skin repair after injury and may contribute to age-associated alterations in wound repair.

Ibrahim et al. (16) studied the post-mortem expression of miR-21 and miR-205 on incisional wounds on a cohort of 18 female albino rats. The animals were injured and then sacrificed, and divided into three groups. In the first group, the samples were taken immediately after death, in the second group after 24 h, and in the third one after 48 h. The researchers analyzed the expression of miRNAs in each sample, observing higher expression in the second group than in the other two. The examined miRNAs were still expressed 48 h after death, although at a lower level. Despite these exciting results, the population studied is too restricted to be considered of meaningful impact and further studies are necessary.

## Materials and Methods

The authors conducted the systematic review according to the Preferred Reporting Items for Systematic Reviews and Meta-Analysis (PRISMA) guideline (56). The quality assessment of this study was evaluated using the Checklist for Systematic Reviews and Research Syntheses recommended by the Joanna Briggs Institute (JBI).

PubMed and Scopus were used as a search engines to find articles published between 1 January 2016 and 1 October 2021, to evaluate the current state of the art regarding wound-age estimation. The Medical Subject Heading (MeSH) thesaurus was used for the following word: “(wound-age estimation) and (wound healing)”; “(wound-age estimation) and (post-mortem)”; “(wound-age estimation) and (miRNA).”

### Inclusion and Exclusion Criteria

The inclusion criteria were: Article in English, Case report; Case series; Original Article; *in vivo* studies (both animals and human); Studies with searching for specific biomarkers.

The exclusion criteria were: Article not in English; Abstract; Poster; Proceedings; Review; Meta-Analysis. The researchers decided to exclude reviews and meta-analysis to give an experimental attitude to the article, analyzing only experimental research.

**Figure 1:**
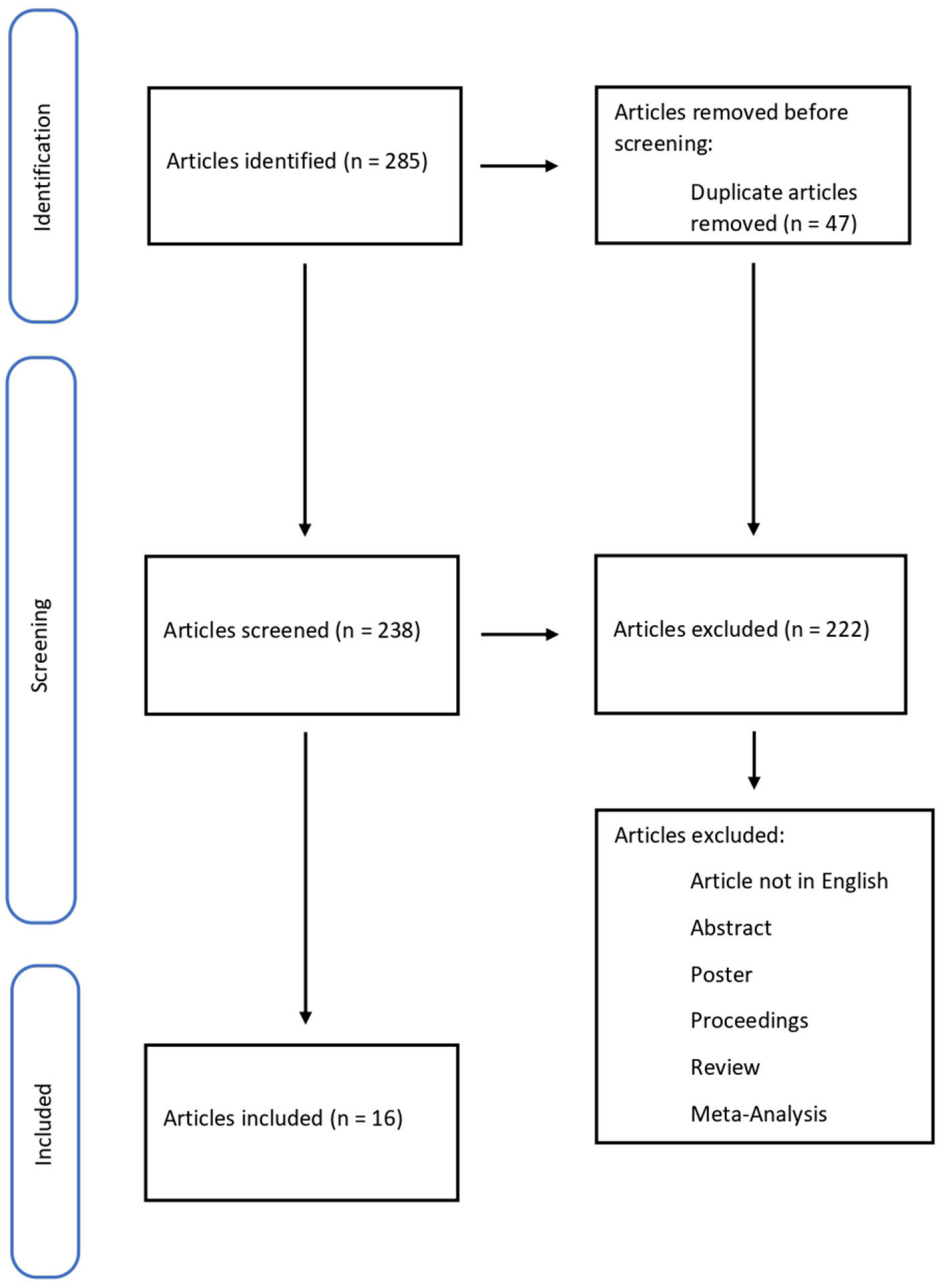
Flow diagram illustrating studies included in and excluded from this systematic review.

The methodology of the search strategy is presented in Figure 1.

### Methodological Evaluation

Methodological evaluation of each study was conducted according to the PRISMA standards, including assessment of bias. Data collection involved study selection and data extraction. Three researchers (S.D.S., M.A.B., E.G.) independently reviewed those documents whose title or abstract appeared to be relevant and selected those who analyzed the wound-age estimation.

### Risk of Bias

This systematic review focuses on articles published in the last 5 years, doing specific research using a few keywords. This topic represents a restricted research field, in which few experiments have been carried out. The limited number of articles available can be a source of error.

## Results

A total of 285 articles were collected, removing 47 duplicates. 238 papers were screened, and 222 did not meet the inclusion criteria; 15 pertinent articles were in Chinese. In conclusion, the systematic review included 16 articles. The studies included in this review were all Original articles (*n* = 16) considering biomarkers for wound-age determination (Table 1).

**Table 1:** Summary of the reviewed literature.

**Biomarker**	**Lesions**	**Sample source**	**Cohort number**	**References**
miR-203, IL-18	Foot ulcers	Male Sprague-Dawley diabetic rats	12	Yuan et al. (57)
MMP2, MMP9, TIMP-1	Human injured skeletal muscle, injured human myocardium, rat heart with vital injuries, and rat heart with injuries inflicted after death	Humans and rats	141 autopsies. Not specified rats number.	Niedecker et al. (58)
Dendritic cells	Stab wounds, incised wounds, surgical wounds, lacerations	Humans	53	Kuninaka et al. (59)
Receptor for advanced glycation end products (RAGE)	Incisional skin wounds	Diabetic rats	Not specified	Ji et al. (60)
iNOS, IL-6	Burned skins	Male albin rats	50	El Noor et al. (61)
mRNA encoding SFRP5, FZD4, Fosl1	Contused muscle	Male Sprague-Dawley rats	78	Zhu et al. (62)
miR-21	Excisional skin wounds	Mice	Not specified	Long et al. (63)
Abhd2, Rael, Asb5, Slfn3, Samd4b, Prr5, Arid5a, Ier3, Cdc40, CD68, Tbx18, Ipo4, Fam210a, Tmem100, Sc65, Legend, Mad212, Rcc1l, Anxa11, Hst6st1, Leprot, Trit1, Polpid3, Lin37, Prrx2, Fbxw4, Prr3, Dclre1b, Impact, Dennd5a, Teme45b, Myg1, Lrrc4l, Rabepk, Rhbdd3	Contused muscle\Rats	Male Sprague-Dawley rats	108	Du et al. (64)
CCL4, CXCL5, IL-1β, IL-6, IL-7	Incisional skin wounds	Male BALB/c mice	72	Gaballah et al. (65)
mRNA encoding TnI, tPA	Contused muscle	Female albino rats	25	Ibrahim et al. (66)
mRNA encoding PUM2, TAB2, GJC1, and CHRNA1	Contused muscle	Male Sprague-Dawley rats	78	Sun et al. (67)
Fosl1	Contused muscle	Male Sprague-Dawley rats	126	Sun et al. (68)
IL-1b, IL-6, TNF-a, IFN-g, MCP-1, CXCL12, VEGF-A, EGF, KGF, pro-col Ia2 and pro-col IIIa1	Excisional skin wounds	Male BALB/c mice	60	Wang et al. (69)
CD14	Skin wounds Postmortem wounds (not specifid type of injury)	Male BALB/c mice Human skin wound	34 97	Yagi et al. (70)
Pax7, MyoD	Contused muscle	Male Sprague-awley rats	40	Tian et al. (71)
3,522 genes	Contused muscle	Male Sprague-Dawley rats	33	Li et al. (72)

## Discussion

The wound-age estimation is still a challenge for forensic pathologists worldwide. They often attend crime scenes, in which bodies show many injuries, whose production time is only conceivable (73). The relevance of this argument encourages more studies on this topic.

In one of the latest reviews in the literature, Li et al. (2) examined the publications made in the years 2010-2016. They stated that there are no standardized and reliable biomarkers to estimate wound-age, despite the study of many molecules. The use of multiple markers could make dating more reliable.

From 2016, as emerged from our literature analysis, only a few studies have been published. All these papers specify a need for further researches.

Yuan et al. (57) investigated the role of miR-203 and IL-8 in the healing process of diabetic foot ulcers in rats. MiR-203 is involved in wound repair and promotes apoptosis, while IL-8 promotes cell proliferation and survival. MiR-203 directly downregulates mRNA expression for IL-18, inhibiting the wound healing process. However, the authors do not evaluate miR-203 or IL-18 expression timing, highlighting only greater miRNA expression in diabetic tissues.

Niedecker et al. (58) studied markers such as matrix metalloproteinases (MMP) 2 and 9 and tissue inhibitors of matrix metalloproteinases 1 (TIMP-1) in human and animal tissues. The authors collected samples of human injured skeletal muscle, injured human myocardium, rat myocardium both with vital and post-mortem injuries. Immunohistochemical investigations showed that these markers did not help wound-age estimation, as the samples did not show positivity (or weak positivity in older wounds).

The research of Kuninaka et al. (59) examined dendritic cells. Dendritic cells (DCs) are cells involved in the immune response and healing the burned skin. The authors collected 53 samples of injured human skin whose production time was known (from a few hours to 21 days). The CD11c and HLA-DRα markers detect the presence of DCs. Wounds aged 4 to 7 days showed low DCs, similar to 9 to 14 days. The samples aged 4-14 days showed the presence of more than 50 DCs per observation field. In the samples aged 17-21 days, this number gradually decreased.

This study indicates that DCs could be a good marker for wound-age estimation, even if the time interval of expression is too broad. Also, Bacci et al. (74) performed a study on DCs cells, measuring their concentration within hours of injury (a short range of time).

Ji et al. (60) made incisional injuries on diabetic mice that they killed after a defined time (6 h to 14 days), finally sampling the lesions. Subsequently, they looked for the expression of the receptor for advanced glycation end products (RAGE) expressed mainly by polymorphonuclear cells. There was an upregulation of RAGEs by 60% between 7 and 10 days after the wounds. However, the authors suggest cautiously interpreting these results, as the diabetic mice used for the study had persistent hyperglycemia from 4 weeks of age and never received treatment.

El-Noor et al. (61) studied the expression of inducible nitric oxide synthase (iNOS) and IL-6 in burned rat skin samples. The authors caused burns on the skin of the mice, sampling them after a defined time (1, 3, 5, 7, 9, 11, 13, 15, and 21 days after the burn). Six hours after the death of the rats, they inflicted another burn, also sampled. The study of markers in ante-mortem wounds revealed that iNOS becomes positive between 3 and 5 days, peaking on day 7, after which it begins to decline. The expression of IL-6 is also time-dependent, starting on day one and peaking on day 3, remaining high until day 5. The positivity begins to decrease from day 7 until it disappears by day 21. In the post-mortem wounds, both markers were weakly positive.

Zhu et al. (62) studied the expression of mRNAs encoding frizzled-related protein 5 (SFRP5), frizzled class receptor 4 (FZD4), and Fos-link antigen 1 (Fosl1) in bruised muscles of rats. These proteins are involved in the healing of injured muscle. The authors monitored their expression for up to 48 h; all mRNAs showed changes, although very different. These trends need further studies to understand if the coding proteins could be considered as useful markers.

Research by Long et al. (63) focused on studying the expression of mir-21 on the skin of female rats with excisional wounds. The authors demonstrated that miR-21 expression improved cutaneous wound repair. However, the purpose of this paper was not to use miR-21 to date the injuries production, so it cannot have forensic application.

Du et al. (64) studied the expression of 35 wound healing-related genes on contused muscle tissue samples from rats through PCR. The authors divided the samples into three groups time-based. Of the 35 genes, 14 showed particular utility in discriminating between the three age groups based on their expression time.

Gaballah et al. (65) studied the expression of five cytokines from the measurement of their RNA through qRT-PCR: chemokine ligand 4 (CCL4), chemokine ligand 5 (CXCL5), interleukin-1 beta (IL-1β), interleukin-6 (IL-6), and interleukin-7 (IL-7). The authors performed incisional wounds on the hamstring muscle of 72 mice, divided into five groups based on the time of wound collection (6, 12, 24, 36, and 48 h post-injury). CXCL5 was the most upregulated molecule with higher expression 6-36 h after injury, such as CCL4 and IL-1β. IL-6, on the other hand, peaked at 6 h post-injury, to significantly decrease at 12 h. IL-7 showed a slow and steady increase over time up to 48 h after injury. Immunohistochemical staining of the post-injury samples showed a gradual increase in intensity around the wound edges. In light of these promising results, this study points out that since post mortem RNA degrades quickly, the application of protein markers is more reliable.

Ibrahim et al. (66) analyzed skeletal troponin I mRNA (TnI) and tissue plasminogen activator (tPA) mRNA expression after inflicting a bruised wound on 25 rats. The researchers divided the rats into five groups based on the injury time. tPA expression levels significantly decreased at 1, 6, and 30 h after contusion. In contrast, TnI expression levels increased at 1 and 6 h post-traumatic, then gradually reduced to normal levels at 24 h, and they assumed significantly lower levels at 30 h after the contusion.

Sun et al. (67) developed an “up, no change or down” system to study the time-dependent expression of PUM2, TAB2, GJC1, and CHRNA1 mRNAs in bruised skeletal muscle of rats. The rats were divided into 12 groups based on the time of wound production. Subsequently, the authors combined the levels of mRNAs. The mRNA levels of PUM2, TAB2, and GJC1 decreased, while CHRNA1 mRNA levels increased. The proposed system can detect the injury time for short periods.

In another research article, Sun et al. (68) such as Zhu et al., studied Fosl1 mRNA and protein in contused skeletal muscle. The researchers divided the rats into different groups (control, contused, and postmortem). They then examined the expression of Fosl1 mRNA and the coding protein in bruised muscle, uninjured contralateral, and postmortem femoral muscle samples. Both mRNA and protein levels of Fosl1 exhibited time-dependent expression during contused skeletal muscle healing. There was no significant difference in Fosl1 protein levels among the postmortem muscle and control specimens, which means that Fosl1 protein levels remained stable in the postmortem samples within 24 h. The study suggests that the expression of Fosl1 mRNA was susceptible to the degree of injury. Fosl1 mRNA could be a promising marker to determine the force that caused the wound but is not stable enough to estimate the duration of the wound.

Wang et al. (69) studied several molecules, including IL-1b, IL-6, TNF-a, IFN-g, MCP-1, CXCL12, VEGF-A, EGF, KGF, pro-col Ia2, and pro-col IIIa1. The authors inflicted skin wounds and subsequently removed them after standardized times. The expression of each molecule was increased over a different time range, depending on their role in wound repair.

Yagi et al. (70) looked for CD14 expression in mouse wounds and postmortem humans. In particular, in humans, they evaluated CD14 in combination with other proteins, such as CD32B and CD68. CD14 appears to be a valuable marker of wound age, 1–5 days postinfliction. However, the combination of several markers increases the sensitivity of the method.

Tian et al. (71) investigated the time-dependent expression of transcription factor 7 (Pax7) and myoblast-determining protein (MyoD) during skeletal muscle wound healing. The experiment consisted of injuring the right limb of rats. Pax7 protein peaked 5 days after injury and subsequently declined, while MyoD mRNA expression peaked 3 days after injury. In conclusion, Pax7 and MyoD expression are time-dependent upregulated during skeletal muscle wound healing, suggesting that they may become potential markers for estimating wound age in skeletal muscle.

Li et al. (72) collected samples of animal injured skeletal muscle taken from the right posterior limb and investigated differentially expressed genes (DEGs) using microarray analyses. A total of 2,844 and 2,298 DEGs involved in muscle repair were identified in the mild and severe contusions, respectively. These DEGs overlap during the early stages after injury (within 48 h). During later stages (at 168 h) there is an expression of only 29 genes. The genes showed time-dependent patterns of expression, which provide a basis for further studies of wound age estimation.

There are not many recent studies on wound-age estimation, probably because of sampling difficulties. Most studies regarded animals but in a low number cohort, summarized in Table 1. Studies on human tissues are often problematic to apply because of ethical requirements (75). Most of the studies also concern protein markers and their mRNA, analyzed by immunohistochemistry, Western-Bloth, and PCR.

From a forensic point of view, it is challenging to select a homogeneous sample of patients due to the difficulty of knowing the lesion's production time. It is essential to know the timing of lesions to experimentally study the expression trend of a potential marker.

Only Kuninaka et al. (59) and Yagi et al. (70) performed studies sampling human wounds with known production time. However, in the studies on rats, the production time was established by the researchers.

## Conclusion

The wound-age estimation is an essential issue in forensic pathology. Pathologists need sensitive and specific markers to conduct an objective assessment and to obtain scientifically and validated evidences. The literature review showed that analysis of miRNA is an innovative field of study with significant potentiality in forensic pathology. The review on the wound-age estimation revealed that research has not progressed in the past 5 years. There are few studies, and almost all of them are at an early stage. On the other hand, the field of wounds' dating in forensic pathology is essential. The main problem in obtaining sufficient data for validation is the difficulty of collecting adequate and homogeneous samples. Several studies recommend the use of multiple methods to get reliable results. In our opinion, the challenge is to understand how to standardize the samples' selection to obtain reliable experimental data. This observation represents a necessary prerequisite to planning further clinical trials.

## Author Contributions

SD, MF, and EG: analyzed the literature. SD and MB: writing. LC and RL: review and editing. GB and SV: language supervision. All authors have read and agreed to the published version of the manuscript.

## Conflict of Interest

The authors declare that the research was conducted in the absence of any commercial or financial relationships that could be construed as a potential conflict of interest. The reviewer AM declared a shared affiliation, with no collaboration, with one of the authors SV to the handling editor at the time of the review.

## Publisher's Note

All claims expressed in this article are solely those of the authors and do not necessarily represent those of their affiliated organizations, or those of the publisher, the editors and the reviewers. Any product that may be evaluated in this article, or claim that may be made by its manufacturer, is not guaranteed or endorsed by the publisher.
